# *Endarachne binghamiae* Extract Alleviates Colitis by Suppressing NLRP3 Inflammasome Activation via Regulation of NOX–iNOS Crosstalk

**DOI:** 10.3390/ijms27062674

**Published:** 2026-03-14

**Authors:** Sang Seop Lee, Sang Hoon Lee, So Yeon Kim, Bong Ho Lee, Yung-Choon Yoo

**Affiliations:** 1 Department of Microbiology, College of Medicine, Konyang University, Daejon 32992, Republic of Korea; wgd.aria@gmail.com (S.S.L.); hoonbin1018@gmail.com (S.H.L.); sksthdus09@hanmail.net (S.Y.K.); 2 Department of Chemical and Biological Engineering, College of Engineering, Hanbat National University, Daejon 34158, Republic of Korea; lbh011@hanbat.ac.kr

**Keywords:** IBD, iNOS-NOX crosstalk, NLRP3, p65 RELA, brown algae

## Abstract

Inflammatory bowel disease (IBD) is triggered by genetic predisposition and chronic inflammation, with aberrant activation of the innate immune complex NLRP3 inflammasome playing a pivotal role in its pathogenesis. In this study, we investigated the effects of a hot water extract from the brown alga *Endarachne binghamiae* (EB-WE) on the inhibition of NLRP3 inflammasome activation, with a focus on its antioxidant properties, in various inflammation models. In bone marrow-derived macrophages (BMDMs), NLRP3 inflammasome activation was induced using LPS and ATP, and EB-WE pretreatment (100, 200 µg/mL) significantly reduced the secretion of IL-1β and IL-18. Confocal immunofluorescence analysis further confirmed that EB-WE suppressed the formation of the NLRP3-ASC/caspase-1 complex. Furthermore, the in vivo anti-IBD efficacy of EB-WE was assessed using a DSS-induced mouse model, in which colonic inflammation and NLRP3-mediated responses were prominent. Oral administration of EB-WE (2 or 5 mg/day) markedly ameliorated clinical symptoms, such as weight loss, diarrhea, and rectal bleeding, and significantly reduced the disease activity index (DAI). EB-WE also decreased serum pro-inflammatory cytokine levels and the expression of NLRP3 inflammasome-related molecules in colon tissue at both the gene and protein levels. In both BMDMs and the IBD mouse model, we further analyzed the upstream regulatory pathway involving NOX2-iNOS. EB-WE efficiently inhibited the activation of the NOX-iNOS axis and NF-κB phosphorylation, thereby alleviating inflammasome activation associated with DSS-induced oxidative stress and neutrophil/macrophage infiltration. Collectively, these results demonstrate that EB-WE effectively suppresses the formation and activation of the NLRP3 inflammasome by modulating the NOX-iNOS axis and the NF-κB pathway via antioxidant mechanisms. These findings suggest that EB-WE holds promise as a novel marine-derived natural therapeutic agent for the treatment of chronic inflammatory diseases.

## 1. Introduction

Inflammatory bowel disease (IBD) is a severe chronic inflammatory disorder in which the innate immune complex, the NLRP3 inflammasome, plays a central role in disease pathogenesis [1]. Under inflammatory conditions, NLRP3 forms a multiprotein complex with the adaptor ASC and caspase-1, leading to the maturation and secretion of pro-inflammatory cytokines such as IL-1β and IL-18, thereby driving potent immune responses and tissue damage [2,3]. In animal models of colitis, excessive activation of NLRP3 aggravates intestinal inflammation, whereas genetic deletion of NLRP3 markedly attenuates the symptoms and tissue injury induced by DSS [4]. These findings are consistent in patients with Crohn’s disease and ulcerative colitis, further supporting the pivotal role of NLRP3 inflammasome in IBD pathogenesis [5,6]. Therefore, inhibition of the NLRP3 inflammasome pathway has emerged as a promising strategy to prevent the progression of IBD and metabolic diseases.

Traditionally, NLRP3 inflammasome activation has been linked to upstream inflammatory signaling pathways such as TLR4/MyD88/AP1 or apoptotic cascades including BAX/cytochrome c/caspase-3 [7,8]. However, recent studies have revealed that NLRP3 activation can be directly triggered not only by receptor-dependent mechanisms but also fundamentally by increased intracellular oxidative stress [9]. Elevated intracellular ROS activates CaMKII through enhanced calcium influx and calmodulin signaling, independently of TAK1 [10]. This leads to the phosphorylation and subsequent degradation of IκBα, allowing NF-κB (p65/RelA) to translocate into the nucleus, where it promotes the transcriptional priming of NLRP3 and TXNIP. The elevated expression of TXNIP further facilitates NLRP3 activation by linking redox imbalance to inflammasome assembly [11].

This oxidative stress-driven inflammation is orchestrated through the depletion of NADPH, primarily regulated by NADPH oxidases (NOXs) [12,13]. The NOX/DUOX (Dual oxidase) family localized on the plasma and endosomal membranes utilizes NADPH to generate superoxide (O_2_^−^) as a means of host defense [14]. The superoxide is then dismutated to H_2_O_2_ by SOD [15]. Chronic NOX activation leads to a rapid reduction in intracellular NADPH levels, thereby decreasing the reduced forms of major antioxidant molecules such as CAT, GSH, GRX, TRX, PRDX, and NQO [16]. This redox imbalance facilitates IκB ubiquitination and p65 nuclear translocation, while also enhancing the intracellular activity of NOX4—an isoform distinguished by its ability to directly produce hydrogen peroxide (H_2_O_2_) rather than superoxide. Unlike other NOX enzymes, NOX4 is constitutively active and typically upregulated in later stages of cellular stress, contributing to sustained oxidative signaling within organelles such as the ER and mitochondria [17]. Nuclear p65 upregulates the expression of COX2, MMP9, various inflammatory cytokines, and inducible nitric oxide synthase (iNOS) [18]. Although H_2_O_2_ does not directly react with NO to form peroxynitrite (ONOO^−^), its sustained accumulation depletes major antioxidant defenses such as NADPH/GSH, GRX/TRX/PRDX, and catalase. This redox imbalance impairs SOD activity, allowing intracellular superoxide (O_2_^−^) to accumulate and react with NO, thereby indirectly accelerating ONOO^−^ formation [19]. ONOO^−^ induces nitration and oxidation of specific tyrosine and cysteine residues in NLRP3, ASC, and caspase-1, directly mediating NLRP3 activation in the later phase [20]. The reduction mechanism for this process is primarily governed by the thioredoxin system; however, NOX-mediated NADPH depletion impairs thioredoxin reductase (TR) activity via TXNIP activation, resulting in increased cellular vulnerability [21].

Recent conceptual frameworks have proposed that NOX–NOS crosstalk represents a fundamental redox signaling axis driving multi-organ inflammatory and metabolic dysregulation [22]. In this context, intestinal inflammation may represent one organ-specific manifestation of this broader redox network. From this perspective, we investigated the IBD-suppressive effects of a hot water extract of the brown alga *Endarachne binghamiae* (EB-WE), known for its reported antioxidant, anti-inflammatory, and anti-cancer properties. Our previous studies demonstrated that EB-WE potently ameliorates LPS-induced acute pneumonia [23] and significantly improves MASLD induced by a high-fat diet through metabolic and antioxidant mechanisms [24]. Based on UHPLC-QTOF-LC-MS/MS and in silico analyses from our prior research, pyropheophorbide (a chlorophyll a-derived metabolite) and loliolide (a benzofuran compound) were tentatively identified as putative bioactive constituents of EB-WE [24]. Given that both compounds have previously been associated with antioxidant and anti-inflammatory activities, these findings may support the hypothesis that EB-WE exerts broad efficacy in ameliorating both inflammatory and metabolic dysfunctions, likely through a common upstream mechanism involving redox regulation and antioxidant activity [25,26].

Based on this hypothesis, we evaluated the effects of EB-WE in a BMDM (LPS + ATP-induced inflammasome activation) model and a DSS-induced IBD mouse model. The present study aims to elucidate the NLRP3 inflammasome-inhibitory effects and underlying mechanisms of EB-WE, with a particular focus on redox-regulated inflammatory signaling, thereby providing insights into the therapeutic potential of marine algal natural products for inflammatory diseases.

## 2. Results

### 2.1. Phytochemical Profile of EB-WE

**Figure 1:**
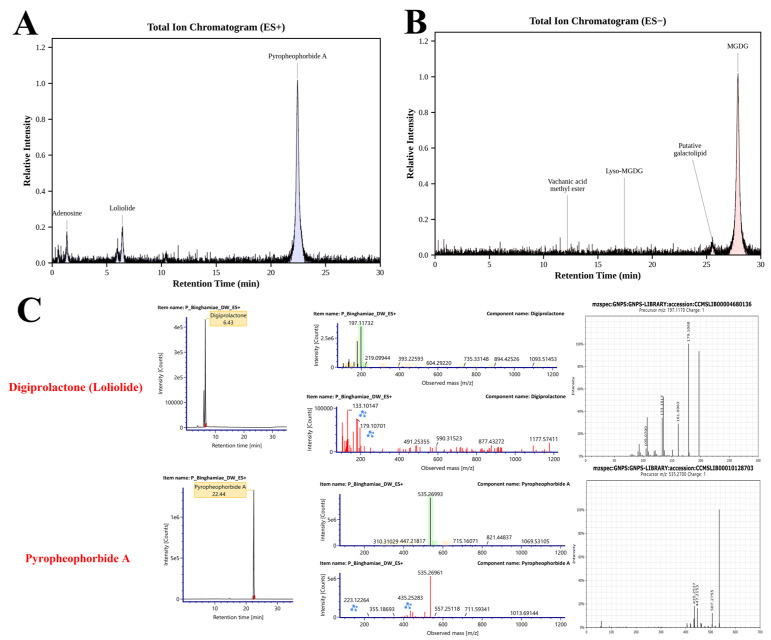
LC-MS/MS-based phytochemical profiling of *Endarachne binghamiae* hot water extract (EB-WE). (**A**) Total ion chromatogram (TIC) in positive ionization mode (ES+). (**B**) TIC in negative ionization mode (ES−). Major peaks are annotated with putatively identified compound names. (**C**) Comparison of MS/MS spectra for two selected major compounds between reference standards and GNPS library matches. Colors and symbols were used to distinguish the experimental and GNPS-matched features in the spectral overlays.

LC-MS/MS-based metabolite profiling of EB-WE revealed distinct chemical signatures in both positive and negative ionization modes (Figure 1, Section 4.1). In ES+ mode, pyropheophorbide A was identified as the dominant compound, while monogalactosyldiacylglycerol (MGDG) predominated in ES− mode, consistent with typical brown algal lipid profiles. A total of 12 compounds were putatively identified through GNPS molecular networking analysis. Based on the selection criteria described in Section 4.1, pyropheophorbide A and loliolide were prioritized as major putative bioactive constituents for further investigation.

### 2.2. Inhibition of NLRP3 Inflammasome Formation by EB-WE in BMDMs

**Figure 2:**
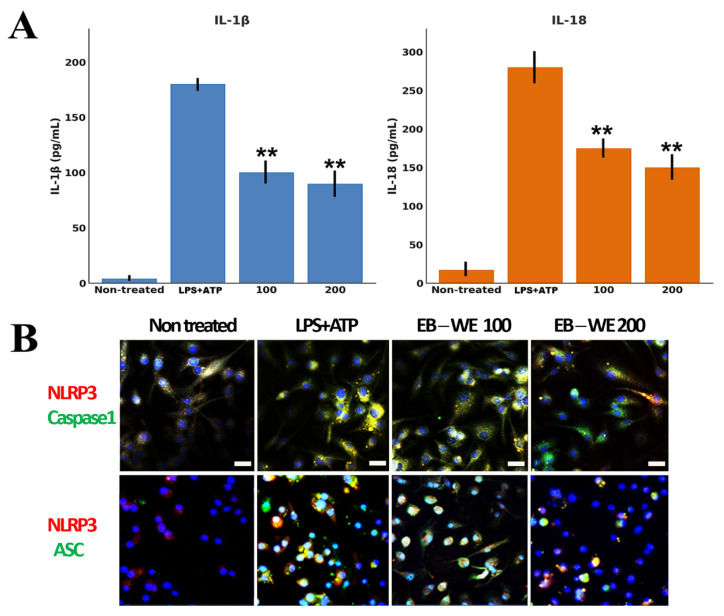
Downregulation of pro-inflammatory cytokines and inhibition of NLRP3 inflammasome formation by EB-WE. (**A**) BMDMs were primed with LPS (1 µg/mL, 4 h) and stimulated with ATP (5 mM, 30 min), with or without EB-WE pretreatment (100 or 200 µg/mL). ELISA analysis showed that LPS + ATP markedly increased IL-1β and IL-18 secretion, while EB-WE pretreatment significantly reduced both cytokines in a dose-dependent manner. (**B**) Confocal immunofluorescence analysis of NLRP3 inflammasome complex formation. NLRP3 is shown in red, caspase-1 or ASC in green, and nuclei are stained with DAPI (blue). Yellow signals indicate colocalization. LPS + ATP stimulation induced robust co-localization of NLRP3 with both caspase-1 and ASC, consistent with inflammasome assembly and pyroptotic speck formation. EB-WE pretreatment markedly reduced NLRP3 co-localization with caspase-1 and ASC, indicating inhibition of inflammasome assembly. Scale bar = 20 µM. These results suggest that EB-WE suppresses both the activation and assembly of the NLRP3 inflammasome, thereby inhibiting pro-inflammatory cytokine maturation and pyroptosis in macrophages. ** *p* < 0.01 vs. LPS + ATP Data are expressed as mean ± SD (*n* = 3).

To investigate the inhibitory effect of EB-WE on NLRP3 inflammasome activation, bone marrow-derived macrophages (BMDMs) were stimulated with LPS and ATP. Upon activation, BMDMs released large amounts of IL-1β and IL-18. However, pretreatment with EB-WE (100 and 200 µg/mL) significantly and dose-dependently reduced the secretion of IL-1β and IL-18 in response to LPS + ATP stimulation (Figure 2A). Specifically, IL-1β secretion in the EB-WE 200 µg/mL group was reduced to approximately 50% of the inflammasome-activated control, with a similar suppression observed for IL-18 (*p* < 0.05). In addition, confocal immunofluorescence staining was performed to visualize the formation of NLRP3, ASC, and caspase-1 complexes (Figure 2B; see also Figure S1 for complete images). In the control BMDMs, robust co-localization of NLRP3 and ASC was observed, indicative of inflammasome speck formation, whereas EB-WE treatment markedly decreased the formation of NLRP3-ASC complexes. Similarly, co-localization of NLRP3 and caspase-1 was also significantly reduced in the EB-WE-treated group, providing visual evidence that EB-WE effectively inhibits inflammasome assembly in macrophages under LPS + ATP stimulation. Collectively, these results indicate that EB-WE suppresses both the assembly and activation of the NLRP3 inflammasome in macrophages, thereby inhibiting the maturation and secretion of IL-1β and IL-18.

### 2.3. Western Blot Analysis of NLRP3-Related Proteins in the BMDM Model

**Figure 3:**
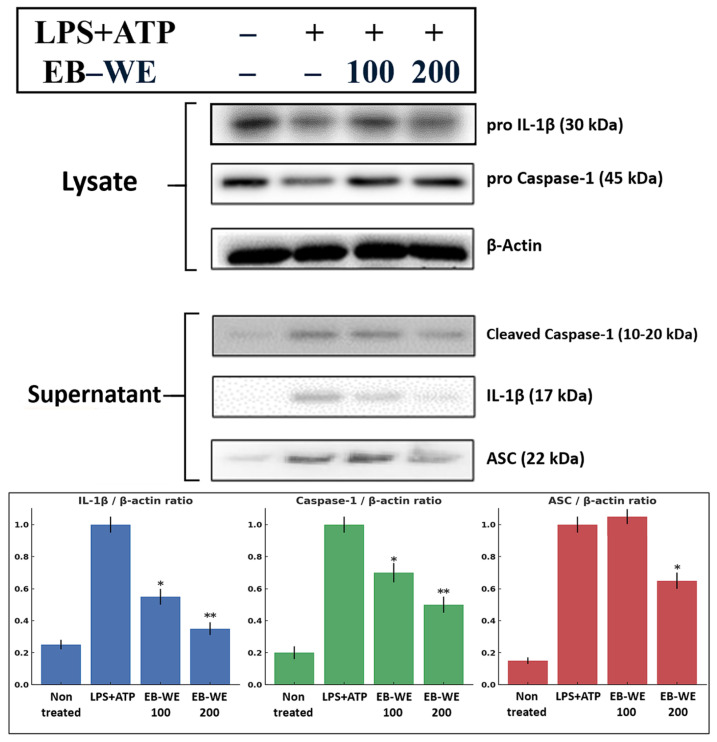
Western blot analysis in BMDMs. BMDMs were primed with LPS (1 µg/mL, 5 h) and stimulated with ATP (5 mM, 30 min), with or without EB-WE pretreatment (100 or 200 µg/mL). Cell lysates and culture supernatants were analyzed for pro-IL-1β (30 kDa), pro-caspase-1 (45 kDa), mature caspase-1 (p10), mature IL-1β (p17), and ASC (22 kDa) by immunoblotting. β-actin served as the loading control. Densitometric quantification is shown for IL-1β, caspase-1, and ASC (normalized to β-actin; mean ± SD, *n* = 3). LPS + ATP markedly increased pyroptotic protein release, while EB-WE reduced mature IL-1β and ASC levels in the supernatant, indicating inhibition of NLRP3 inflammasome activation. * *p* < 0.05, ** *p* < 0.01 vs. LPS + ATP group. Data are expressed as mean ± SD (*n* = 3).

To further validate the ELISA and immunohistochemical findings, Western blotting was performed to assess the effects of EB-WE on the expression and secretion of key inflammatory proteins at the protein level. The results supported the trends observed in Section 2.1. In control BMDMs activated with LPS + ATP, the expression of pro-caspase-1 was markedly increased, and substantial amounts of mature IL-1β (p17) and ASC proteins were released into the culture supernatant, reflecting effective pyroptosis via NLRP3 inflammasome activation. In contrast, EB-WE treatment resulted in increased intracellular levels of pro-IL-1β and pro-caspase-1, while the amounts of mature IL-1β and ASC released into the supernatant were significantly reduced compared to the control (Figure 3). These findings indicate that EB-WE exerts anti-inflammatory effects by inhibiting the formation of the NLRP3 inflammasome complex.

### 2.4. EB-WE Attenuates ROS-Mediated NLRP3 Inflammasome Activation in BMDMs

**Figure 4:**
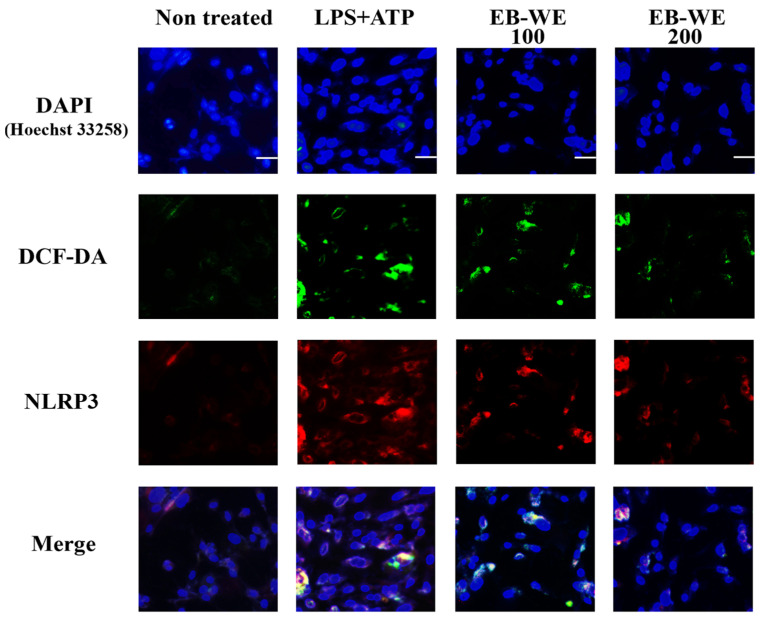
Confocal immunofluorescence images showing the intracellular localization of ROS (DCF-DA, green), NLRP3 (red), and nuclei (DAPI, blue) in bone marrow-derived macrophages (BMDMs). Cells were primed with LPS (1 µg/mL, 5 h) and stimulated with ATP (5 mM, 30 min), with or without EB-WE pretreatment (100 or 200 µg/mL, 24 h). LPS + ATP stimulation induced strong DCF-DA fluorescence and perinuclear NLRP3 clustering, which were both markedly reduced by EB-WE. Merged images reveal co-localization of ROS and NLRP3 in the activated state, potentially indicating that its anti-inflammatory effect is linked to antioxidant activity. Scale bar = 20 µM.

Based on our previous findings demonstrating the antioxidant activity of EB-WE [23,24], we further investigated the interplay between intracellular ROS generation and NLRP3 inflammasome activation in BMDMs. Live-cell staining with DCF-DA (green) was first performed to detect ROS, followed by fixation and immunofluorescence staining for NLRP3 (red). Nuclei were counterstained with DAPI (blue), and all images were acquired using confocal microscopy. As shown in Figure 4, LPS + ATP treatment markedly increased both ROS accumulation and perinuclear clustering of NLRP3 compared to non-treated controls. Notably, EB-WE pretreatment (100 or 200 µg/mL) led to a pronounced reduction in DCF-DA fluorescence and NLRP3 expression, indicating effective suppression of ROS-mediated inflammasome activation. Merged images clearly demonstrated spatial overlap of ROS and NLRP3 in the LPS + ATP group, which was decreased upon EB-WE treatment. Given the close association between ROS and NLRP3 activation [27], these results imply a potential link between the antioxidant and anti-inflammatory effects of EB-WE.

### 2.5. Anti-Inflammatory Effects of EB-WE in the IBD Mouse Model

**Figure 5:**
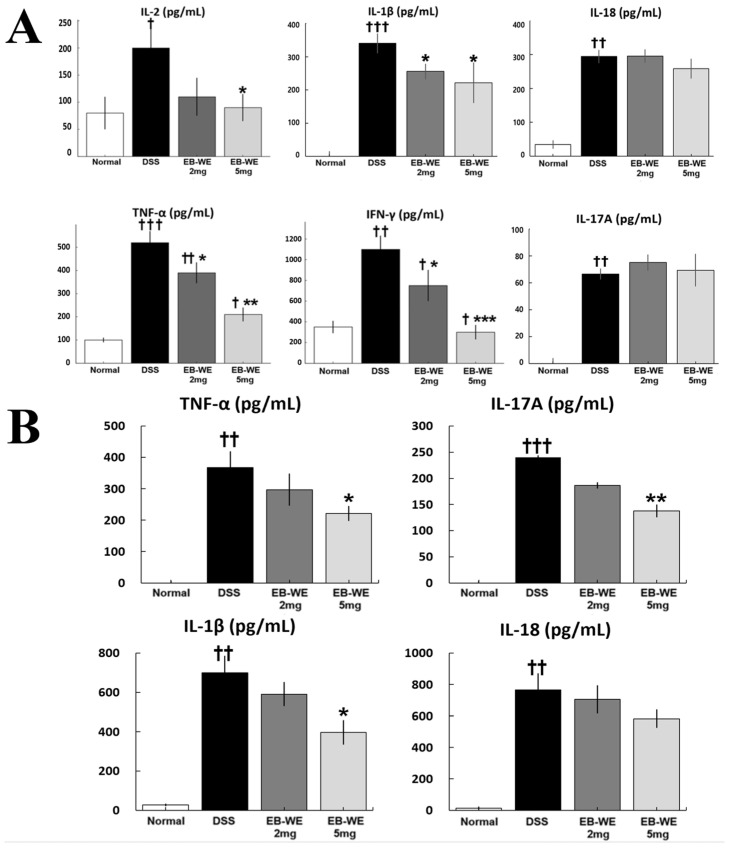
EB-WE suppresses systemic and local inflammation in DSS-induced colitis. (**A**) Serum cytokine levels of IL-2, IL-1β, IL-18, TNF-α, IFN-γ, and IL-17A were measured by ELISA. DSS significantly increased all six pro-inflammatory cytokines, while EB-WE administration, especially at 5 mg/mice, markedly reduced IL-2, IL-1β, TNF-α, and IFN-γ levels. (**B**) Colonic cytokine levels were similarly elevated by DSS, indicating strong mucosal inflammation. EB-WE treatment significantly reduced TNF-α, IL-1β, and IL-17A expression in a dose-dependent manner. † *p* < 0.05, †† *p* < 0.01, ††† *p* < 0.001 vs. Normal; * *p* < 0.05, ** *p* < 0.01, *** *p* < 0.001 vs. DSS.

As shown in Figure 5A, DSS administration significantly elevated the serum levels of multiple pro-inflammatory cytokines, including IL-2, IL-1β, IL-18, TNF-α, IFN-γ, and IL-17A, confirming the presence of systemic inflammation. Notably, EB-WE treatment effectively and dose-dependently reduced these cytokines, with the 5 mg/mice group showing the most consistent anti-inflammatory effects. In particular, IL-2, IL-1β, TNF-α, and IFN-γ levels were significantly decreased compared to the DSS group, indicating suppression of both Th1 and general inflammatory responses.

In addition, to further validate the local anti-inflammatory effects of EB-WE, cytokine levels in colon tissues were assessed (Figure 5B). DSS markedly elevated TNF-α, IL-1β, IL-18, and IL-17A concentrations in the colon, consistent with mucosal inflammation. EB-WE treatment significantly attenuated the expression of TNF-α, IL-1β, and IL-17A, particularly at the 5 mg/mice dose (* *p* < 0.05 or ** *p* < 0.01 vs. DSS), indicating local immunomodulatory activity within the colonic microenvironment. These findings collectively demonstrate that EB-WE confers both systemic and tissue-level anti-inflammatory protection in DSS-induced colitis.

### 2.6. Histological and Clinical Changes in the IBD Model

**Figure 6:**
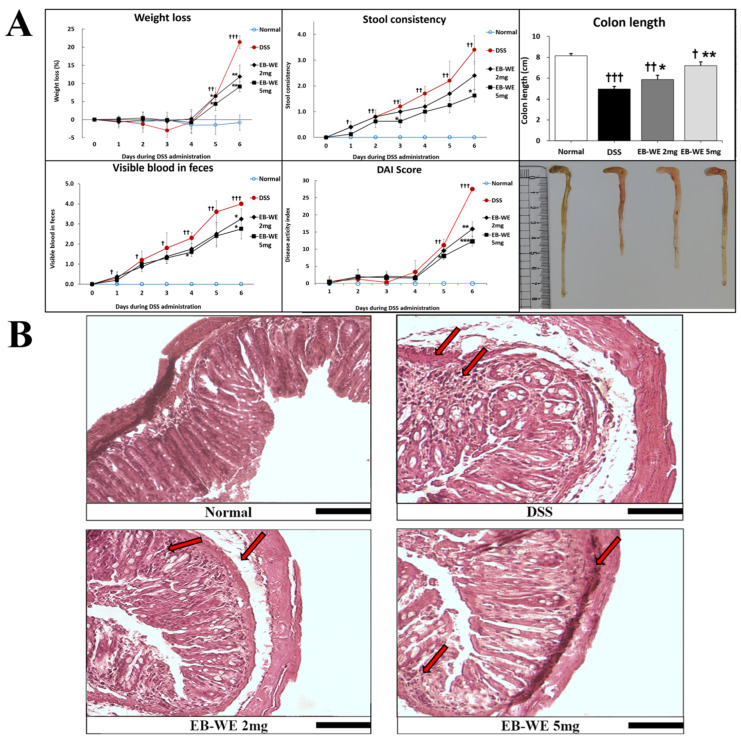
Anti-IBD effects of EB-WE in colon tissue. (**A**) EB-WE treatment significantly alleviated DSS-induced clinical symptoms, including body weight loss, increased stool consistency scores, visible blood in feces, elevated Disease Activity Index (DAI) scores, and colon shortening. The 5 mg/mice group exhibited the most pronounced improvements across all parameters, indicating a dose-dependent therapeutic effect of EB-WE. (**B**) Representative histological images of colon tissue stained with H&E. DSS treatment induced severe epithelial disruption, submucosal edema, and extensive inflammatory cell infiltration (indicated by arrows). EB-WE administration mitigated these pathological features in a dose-dependent manner. The 5 mg/mice group demonstrated nearly intact mucosal and crypt structures, comparable to the normal group. Scale bar = 100 µM. † *p* < 0.05, †† *p* < 0.01, ††† *p* < 0.001 vs. Normal; * *p* < 0.05, ** *p* < 0.01, *** *p* < 0.001 vs. DSS.

As shown in Figure 6A, DSS administration significantly increased weight loss, stool consistency scores, visible blood in feces, and overall Disease Activity Index (DAI) over time, clearly indicating the progression of colitis. In contrast, EB-WE treatment markedly attenuated these DSS-induced clinical symptoms in a dose-dependent manner. The 5 mg/mice group showed the most pronounced improvement across all parameters, with significantly lower weight loss, improved stool consistency, reduced fecal bleeding, and substantially decreased DAI scores (** *p* < 0.01 to *** *p* < 0.001 vs. DSS), suggesting a strong protective effect against DSS-induced colitis.

Histological evaluation further supported these findings (Figure 6B). In the DSS group, severe mucosal damage, destruction of intestinal villi, marked submucosal edema, and massive inflammatory cell infiltration were observed. In the EB-WE 2 mg/mice group, some mucosal damage and inflammatory infiltration remained, but pathological features were notably improved compared to the DSS-only group. Particularly, the EB-WE 5 mg/mice group exhibited near-complete preservation of mucosal and villous structures, with minimal inflammatory signs, closely resembling the normal histological architecture. These results indicate that EB-WE effectively and dose-dependently ameliorates both clinical and histological features of DSS-induced colitis.

### 2.7. EB-WE Suppresses Inflammatory and Apoptotic Signaling While Preserving Epithelial Barrier Integrity in DSS-Induced Colonic Tissue

**Figure 7:**
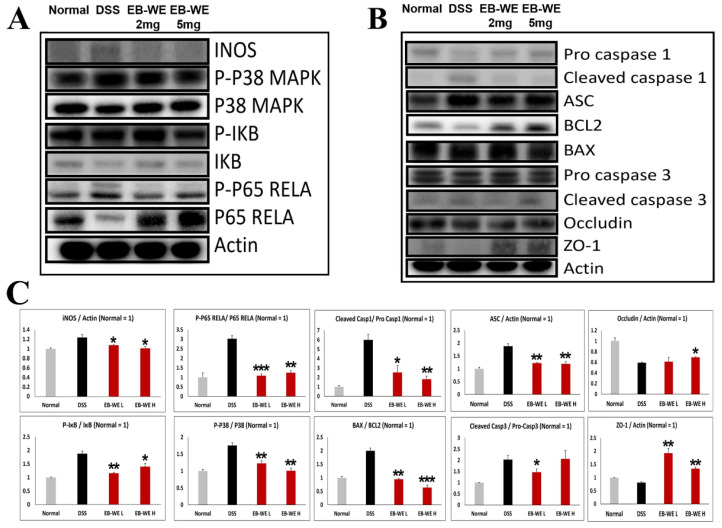
EB-WE suppresses inflammatory signaling, NLRP3 inflammasome activation, and apoptosis in colon tissue of DSS-induced IBD mice. (**A**) Representative Western blot analysis of inflammatory signaling proteins (iNOS, p-p38 MAPK, p38 MAPK, p-IKB, IKB, p-p65 RELA, p65 RELA) in colon tissue. (**B**) Representative blots for inflammasome (pro-caspase-1, cleaved caspase-1, ASC), apoptotic (BCL2, BAX, pro-caspase-3, cleaved caspase-3), and barrier-related proteins (Occludin, ZO-1). Actin was used as a loading control. (**C**) Densitometric quantification of the Western blot bands shown in (**A**,**B**), presented as the relative expression or phosphorylation ratio of each protein normalized to actin or the corresponding total protein, with the normal group set to 1. Colon tissues were collected from normal (Sham), DSS-treated, and EB-WE–treated (2 and 5 mg/mice) groups. * *p* < 0.05, ** *p* < 0.01. *** *p* <0.001 vs. DSS. Data are expressed as mean ± SD (*n* = 3).

To further elucidate the protective mechanisms of EB-WE in vivo, Western blot analysis was performed on colon tissues from DSS-induced IBD mice. As shown in Figure 7, the DSS group exhibited significant upregulation of inflammatory signaling proteins, including iNOS, phosphorylated p38 MAPK, p-IKB, and p-p65, along with activation of the NLRP3 inflammasome pathway, as evidenced by increased levels of ASC and cleaved caspase-1. Pro-apoptotic markers (BAX, cleaved caspase-3) were also elevated, while anti-apoptotic BCL2 and tight junction proteins (Occludin, ZO-1) were markedly reduced.

Conversely, EB-WE treatment (2 or 5 mg/mice) dose-dependently suppressed these inflammation- and apoptosis-related proteins, and it restored the expression of tight junction proteins. These results collectively indicate that EB-WE effectively inhibits both inflammatory signaling and NLRP3 inflammasome activation, while preserving epithelial barrier integrity in DSS-induced colonic tissue.

### 2.8. Modulation of Redox Regulatory Proteins by EB-WE in Colon Tissue of the IBD Model

**Figure 8:**
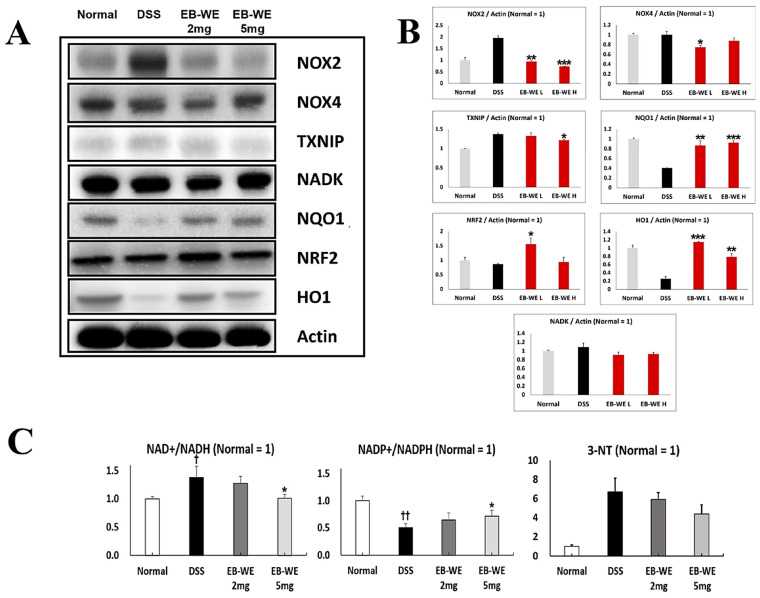
EB-WE modulates redox-regulating proteins and redox cofactor ratios in colon tissue of DSS-induced IBD mice. (**A**) Representative Western blot analysis of pro-oxidant (NOX2, NOX4, TXNIP) and antioxidant (NQO1, NRF2, and HO-1) proteins with Actin as the loading control. (**B**) Quantitative analysis of protein expression normalized to Actin. (**C**) Ratios of NAD+/NADH and NADP+/NADPH measured in colon tissues, and serum 3-nitrotyrosine (3-NT) levels as an index of peroxynitrite-mediated nitrosative stress, from normal, DSS-treated, and EB-WE-treated (2 and 5 mg/mice) groups. † *p* < 0.05, †† *p* < 0.01 vs. Normal; * *p* < 0.05, ** *p* < 0.01, *** *p* < 0.001 vs. DSS. Data are expressed as mean ± SD (*n* = 3).

To elucidate the antioxidant mechanisms underlying the anti-inflammatory effects of EB-WE, we performed Western blot analyses targeting key redox-regulating proteins in colon tissue (Figure 8). DSS administration markedly upregulated the expression of NOX2, NOX4, and TXNIP, reflecting increased oxidative stress in the inflamed colon. In contrast, EB-WE treatment dose-dependently reduced the levels of these pro-oxidant proteins. Notably, EB-WE also restored the expression of NQO1, NRF2, and HO-1 antioxidant defense proteins that were suppressed by DSS challenge. These results suggest that EB-WE attenuates colonic oxidative stress not only by inhibiting ROS-generating enzymes (NOX2, NOX4, TXNIP) but also by activating the cellular antioxidant response (NQO1, NRF2, HO-1). Collectively, these data demonstrate that the redox-modulating effects of EB-WE contribute substantially to its anti-inflammatory actions in DSS-induced IBD.

In addition, to understand the impact of EB-WE on cellular redox balance, we analyzed the expression levels of NAD+/NADH and NADP+/NADPH ratios in colon tissues. The DSS-induced rise in the NAD+/NADH ratio likely reflects a shift toward enhanced glycolytic and mitochondrial oxidative flux under inflammatory stress [28,29], whereas the concomitant drop in NADP+/NADPH indicates exhaustion of the principal reductive reserve needed for glutathione and thioredoxin-dependent detoxification [28,30]. By partially normalizing both cofactor ratios, EB-WE not only tempers excessive pro-oxidant metabolism but also replenishes the reducing power essential for phase II antioxidant enzymes.

In parallel, the downregulation of TXNIP relieves thioredoxin inhibition, synergizing with the upregulation of NQO1, NRF2 and HO-1 to bolster cellular defenses (Figure 8A,B). The unchanged NADK levels, despite restored NADP+/NADPH, suggest that EB-WE may activate alternative NADPH-regenerating pathways such as glucose-6-phosphate dehydrogenase or malic enzyme instead of directly modulating NADK expression.

Finally, the rise in NAD+ availability may further trigger sirtuin-mediated deacetylation events, promoting mitochondrial biogenesis and anti-inflammatory gene expression. Altogether, these coordinated changes in cofactor balance, enzyme expression, and redox-sensitive signaling highlight EB-WE as a multifaceted modulator of metabolic and antioxidant networks in inflamed colon tissue. To further validate the systemic nitrosative stress burden, serum 3-nitrotyrosine (3-NT) levels were measured as a direct biomarker of peroxynitrite (ONOO^−^)-mediated protein nitration. DSS administration markedly elevated serum 3-NT relative to normal controls, confirming excessive in vivo ONOO^−^ generation. EB-WE treatment dose-dependently reduced 3-NT levels, the 5 mg/mice dose achieving a statistically significant decrease (Figure 8C). These findings directly link the NOX–iNOS axis suppression observed at the protein level to a measurable attenuation of nitrosative modification in vivo, substantiating the mechanistic model proposed in the present study.

## 3. Discussion

### 3.1. ROS and NLRP3 Inflammasome: Priming and Oligomerization

Recent studies have reported that ROS can activate NF-κB (p65) independently of TAK1 and, by disrupting the thioredoxin reductase pathway, promote the formation of the NLRP3 inflammasome [31]. Upon DSS challenge, colonic epithelial cells and immune cells (including macrophages) activate NOX, which produces large amounts of superoxide (O_2_^−^) and other ROS to defend against pathogens, but this process rapidly depletes intracellular NADPH [32]. As NADPH is the main source of reducing power for the thioredoxin and glutathione antioxidant systems, its depletion severely impairs antioxidant defense, resulting in the loss of redox homeostasis [33]. Accumulated ROS consequently influence intracellular signaling and can activate p65 RelA independently of the canonical TAK1 pathway [34]. Excessive ROS have been shown to accelerate IκBα degradation, promoting NF-κB (p65/p50 complex) nuclear translocation and upregulation of pro-inflammatory cytokines [35]. DSS promotes IBD by activating NOX in colonic epithelial cells and depleting the thioredoxin system [36]. The oxidative environment, together with macrophage and neutrophil infiltration, cooperatively supports NLRP3 inflammasome activation and perpetuates chronic IBD pathology [37]. Indeed, excessive activation of the NF-κB pathway by oxidative stress has been observed in colitis models and is recognized as a major factor aggravating chronic inflammation.

**Figure 9:**
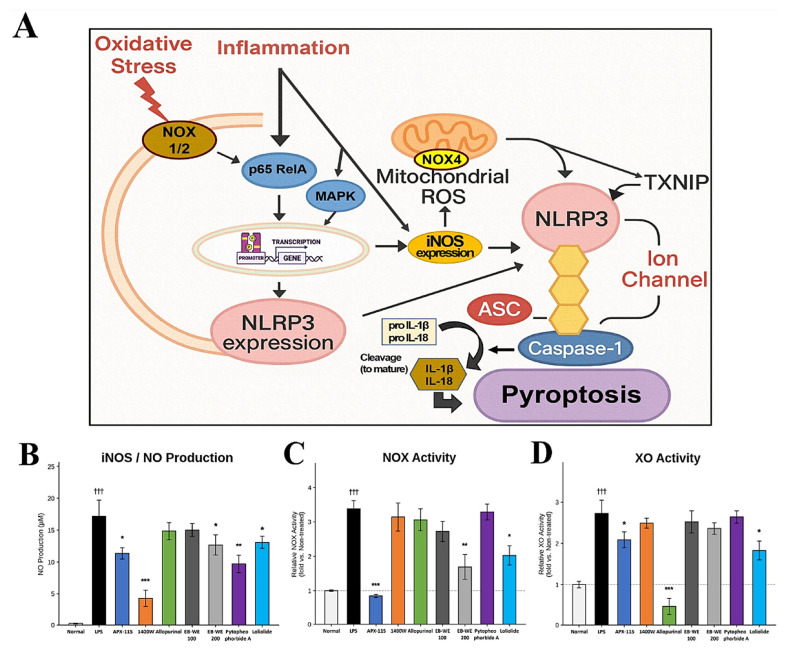
NOX–iNOS Axis as the Central Driver of NLRP3 Inflammasome Activation and Its Functional Validation in LPS-Stimulated Macrophages. (**A**) Schematic diagram illustrating the dual contribution of NOX–ROS and iNOS to NLRP3 inflammasome priming and activation. Both NF-κB (p65) and MAPK signaling pathways mediate transcriptional priming of NLRP3 and iNOS under NOX1/2-derived oxidative stress. NOX4 and iNOS contribute to mitochondrial ROS and peroxynitrite (ONOO^−^) production, facilitating NLRP3 activation through TXNIP dissociation and ion flux, ultimately triggering pyroptosis. (**B**–**D**) Functional validation in LPS-stimulated RAW 264.7 macrophages. (**B**) iNOS activity assessed by Griess reagent-based NO production assay. (**C**) NOX activity measured using a superoxide-based colorimetric assay (E-BC-K815-M). (**D**) XO activity measured using a cell-free inhibitor screening assay (E-BC-D019). Inhibitor controls were APX-115 (10 μM; NOX inhibitor), 1400 W (100 μM; iNOS inhibitor), and allopurinol (100 μM; XO inhibitor). EB-WE concentrations are expressed in μg/mL, and pyropheophorbide A and loliolide were used at 10 μM. Data are expressed as mean ± SEM (*n* = 3). ††† *p* < 0.001 vs. non-treated; * *p* < 0.05, ** *p* < 0.01, *** *p* < 0.001 vs. LPS.

Furthermore, ROS not only enhance the expression of NLRP3 by promoting p65-mediated transcriptional priming, but also support the oligomerization of ASC and activation of caspase-1, thereby facilitating full inflammasome assembly [38,39] (Figure 9A). This dual role of ROS highlights its involvement in both the priming and activation phases of NLRP3 inflammasome signaling. To experimentally validate this pathway in a cellular model, LPS-stimulated RAW 264.7 macrophages were treated with pathway-selective inhibitors and EB-WE constituents. NOX activity, iNOS-derived NO production, and xanthine oxidase (XO) activity were quantified using target-specific biochemical assays (Figure 9B–D). LPS stimulation markedly elevated all three enzyme activities relative to non-treated controls. APX-115 (NOX inhibitor) selectively suppressed NOX activity, 1400 W (iNOS inhibitor) abolished NO production, and allopurinol (XO inhibitor) specifically reduced XO activity, confirming assay selectivity. EB-WE dose-dependently inhibited both NOX and iNOS/NO activities, consistent with its in vivo suppression of NOX2/NOX4 and iNOS protein expression. Pyropheophorbide A preferentially attenuated NO production without substantially affecting NOX or XO activity, suggesting iNOS-directed inhibition. Loliolide inhibited both NOX and XO activities, indicating a dual-oxidase suppression profile consistent with the IKBKB-mediated NF-κB regulatory mechanism predicted by target prediction analysis. Collectively, these cellular data provide functional corroboration of the NOX–iNOS–NLRP3 axis and delineate the distinct mechanistic contributions of each bioactive constituent.

### 3.2. Implications for Inflammation-Focused Mechanistic Analysis

**Figure 10:**
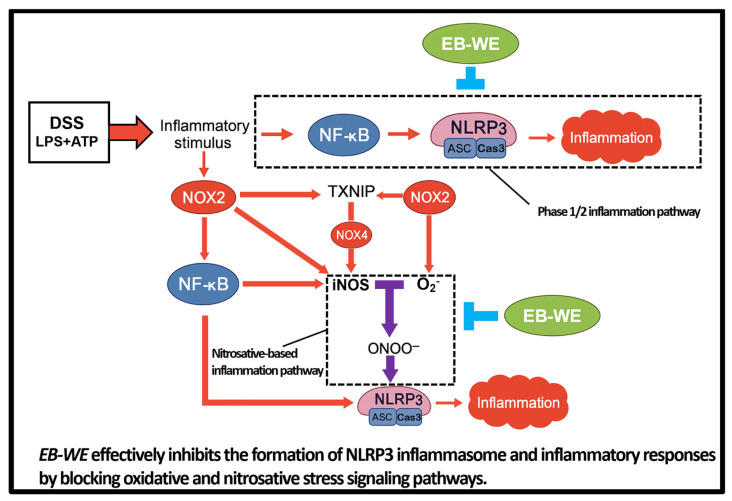
Proposed schematic model illustrating the anti-inflammatory mechanism of EB-WE in DSS-induced colitis.

In this study, we systematically demonstrated that the hot water extract of the brown alga *Endarachne binghamiae* (EB-WE) effectively inhibits NLRP3-mediated inflammatory responses in various experimental models. Briefly, EB-WE suppressed LPS + ATP-induced activation of the NLRP3 inflammasome in macrophages, leading to reduced maturation and secretion of IL-1β and IL-18. In the DSS-induced IBD model, EB-WE ameliorated both inflammatory cytokine production and tissue injury, accompanied by a reduction in the expression of NLRP3 inflammasome-related molecules. These consistent findings, validated at the protein level, suggest that the anti-inflammatory efficacy of EB-WE is primarily mediated through the suppression of the NLRP3 inflammasome pathway. In other words, EB-WE demonstrates the potential to attenuate the progression of chronic inflammatory diseases by inhibiting the excessive activation of the NLRP3 inflammasome, a key contributor to disease pathogenesis (Figure 10).

Inflammatory stimuli such as DSS and LPS/ATP activate NOX2 and NOX4, which promote oxidative stress through H_2_O_2_ production and enhance NF-κB activation. NOX2 also upregulates iNOS expression, leading to excessive peroxynitrite (ONOO^−^) formation via iNOS–H_2_O_2_ interaction. TXNIP acts as a redox-sensitive mediator that links NOX4-derived ROS to NLRP3 inflammasome activation. This cascade culminates in caspase-1 cleavage and IL-1β maturation, amplifying mucosal inflammation. EB-WE treatment inhibits this vicious cycle by suppressing NOX2/NOX4 expression, reducing TXNIP and iNOS levels, and blocking the oxidative/nitrosative stress axis, thereby attenuating NLRP3 inflammasome activation and downstream inflammation.

### 3.3. NOX–iNOS Crosstalk and IBD: Inflammation Driven by Nitrosative Stress

Once NF-κB translocates to the nucleus, transcription of various inflammatory genes is upregulated, with a marked increase in iNOS expression [40]. The high levels of NO generated by iNOS rapidly react with NOX1/2-derived O_2_^−^ to form peroxynitrite (ONOO^−^) [41]. In addition, chronic activation of NOX in macrophages and colonic epithelium has been observed in DSS-induced inflammatory microenvironments [42]. The activation of NF-κB, including p65 RELA, further contributes to increased NOX4 expression [43], and NOX4 continuously produces H_2_O_2_ that, together with iNOS-derived NO, accelerates ONOO^−^ formation [44]. ONOO^−^, a potent reactive nitrogen species (RNS), not only damages cellular components but also nitrates tyrosine residues of proteins, thereby altering their function [45]. In both DSS-induced colitis models and colonic tissues from IBD patients, iNOS overexpression and increased protein nitration (e.g., nitrotyrosine) have been observed concurrently, indicating that excessive ONOO^−^ generation is a key driver of ongoing oxidative injury [46,47].

### 3.4. Oxidative Damage and NLRP3: The Intimate Crosstalk Between Inflammation and Oxidative Stress

This nitrosative stress serves as a crucial trigger for the activation of the NLRP3 inflammasome. Additionally, it has been reported that, as cytosolic thioredoxin becomes oxidized, dissociated TXNIP binds directly to NLRP3, thus functioning as a redox-sensitive mechanism for inflammasome activation [48]. Accordingly, collapse of the thioredoxin system due to NADPH depletion can facilitate the interaction between TXNIP and NLRP3, providing a strong signal for inflammasome activation [49]. Moreover, when peroxynitrite (ONOO^−^) induces nitration of inflammasome components such as NLRP3, ASC, and caspase-1, the resulting modifications favor their oligomerization and activation, thereby promoting the assembly of the NLRP3 inflammasome complex. There is evidence that decomposing or inhibiting the generation of peroxynitrite reduces both priming and activation of the NLRP3 inflammasome, further supporting the direct role of ONOO^−^ in inflammasome activation [50,51].

Overactivation of the NLRP3 inflammasome leads to the processing and massive secretion of mature IL-1β and IL-18 from their precursors, amplifying inflammatory responses in the intestinal mucosa [52]. This, in turn, results in epithelial cell injury, increased intestinal permeability, and reactivation of immune cells, creating a vicious cycle that exacerbates IBD pathology and culminates in pyroptosis [32]. Notably, in chronic DSS-induced colitis models, simultaneous overexpression of NOX, DUOX, and iNOS has been shown to result in the accumulation of excessive ONOO^−^ in local tissues and aggravation of inflammation [53]. This suggests that massive ONOO^−^ generation—stemming from the explosive reaction between NOX-derived ROS and iNOS-derived NO—plays a central role in NLRP3-driven IBD exacerbation [54].

### 3.5. Translational Implications Supported by Human Transcriptomic Data

To validate the relevance of our findings to human pathology, we independently analyzed publicly available transcriptomic data from colonic tissues of IBD patients (GSE16879) [55,56] (Figure S2, Table S1). This dataset revealed a consistent and coordinated upregulation of 13 genes involved in redox and inflammatory signaling—including DUOX1, DUOX2, iNOS (NOS2), CASP1, and IL1B—as well as accessory or amplifying factors such as DUOXA1/2, TANK, NKAPL, and XDH. Notably, LOXL2, a fibrosis-related oxidase, was also significantly elevated. These findings closely mirror the molecular targets modulated by EB-WE in both murine and cellular models of inflammation. Importantly, PRDX6, a cytosolic antioxidant enzyme, was markedly downregulated, suggesting a compromised ROS-buffering capacity in the IBD mucosa. CAMK2N1, an endogenous inhibitor of calcium/calmodulin-dependent kinase II (CaMKII), was also significantly reduced (*p* = 0.00067). Given that DUOX1, DUOX2, and NOX5 are the only NOX isoforms directly activated by Ca^2+^ via EF-hand domains, this suppression of CAMK2N1 suggests enhanced calcium–redox crosstalk and a loss of intrinsic regulation of CaMKII-driven inflammation [22].

Statistical analysis using the Mann–Whitney U test confirmed that all 13 analyzed genes exhibited significant differences (* *p* < 0.05, ** *p* < 0.01, *** *p* < 0.001) between control and IBD tissues. These consistent transcriptomic alterations reinforce the translational relevance of our mechanistic model [57], particularly the notion that oxidative and nitrosative stress synergistically activate the NLRP3 inflammasome through the NOX–iNOS–NLRP3 axis. Collectively, our findings support the therapeutic potential of targeting this axis to mitigate chronic mucosal inflammation in human IBD.

Nevertheless, several limitations of the present study warrant acknowledgment. First, the bioactive effects were primarily characterized using a crude hot water extract; while pyropheophorbide A and loliolide were identified as putative major constituents and individually evaluated in cellular assays, the precise contribution of each compound to the overall in vivo efficacy—and potential synergistic interactions within the extract matrix—remains to be determined. Second, the molecular binding modes of these constituents to NOX2, NOX4, and iNOS have not been directly characterized in the present study. Future investigations employing structure-based molecular docking and activity-based target engagement profiling approaches [58,59] would help delineate the precise binding interactions and selectivity profiles of these compounds, thereby strengthening the mechanistic foundation for their therapeutic development as NOX–iNOS axis inhibitors.

## 4. Materials and Methods

### 4.1. Preparation and Phytochemical Analysis of EB Hot Water Extract

The EB-WE (hot water extract of *Endarachne binghamiae*) used in this study was prepared according to the methods described in our previous report [24]. For phytochemical characterization, compounds putatively identified by UHPLC-QTOF-LC-MS/MS were subjected to a secondary selection based on MS/MS fragmentation pattern matching, previous reports of the compounds in marine algae, and documented antioxidant/anti-inflammatory activities. Subsequently, a third selection was performed using in silico analysis via MetFrag [60], GNPS [61], and SIRIUS [62] platforms, applying a cosine similarity threshold of ≥0.85. Through this workflow, 12 tentative candidate bioactive compounds were identified, with pyropheophorbide and loliolide ultimately selected as the major putative active constituents (Table 1).

### 4.2. BMDM Inflammation Induction and EB-WE Pretreatment—Cell Isolation and Culture

Bone marrow-derived macrophages (BMDMs) were differentiated from three-month-old male C57BL/6 mice. Bone marrow was flushed from the femurs and tibias and cultured in RPMI medium supplemented with 10% fetal bovine serum and L929 cell-conditioned medium (as a source of macrophage colony-stimulating factor) at 37 °C in a 5% CO_2_ incubator for 7 days to induce differentiation of immature monocytes into macrophages. On day 7, BMDMs were harvested and seeded at 1 × 10^6^ cells/well in 12-well plates, allowed to adhere for 24 h, and then used for subsequent experiments.

Cell viability following EB-WE pretreatment (100 and 200 µg/mL) was confirmed by MTT assay prior to all functional experiments; viability remained above 85% at both concentrations, consistent with previously reported cytotoxicity data for the same extract preparation in RAW 264.7 macrophages [23,24]. Accordingly, the pharmacological effects observed in the present study are attributable to genuine anti-inflammatory activity rather than cytotoxic artifact [23].

### 4.3. NLRP3 Inflammasome Activation

To activate the NLRP3 inflammasome in BMDMs, a dual stimulation protocol with LPS and ATP was employed. Cells were primed with LPS (*Escherichia coli* O111:B4, Sigma-Aldrich, St. Louis, MO, USA) at 100 ng/mL for 5 h to induce expression of *Nlrp3* and *Il1b* (priming), followed by ATP (Sigma-Aldrich, St. Louis, MO, USA) at 5 mM for 30 min to trigger NLRP3 inflammasome assembly and caspase-1 activation. For the EB-WE treatment groups, cells were pretreated with EB-WE at final concentrations of 100 or 200 µg/mL one hour before LPS priming. The EB-WE extract was prepared by suspending freeze-dried *Endarachne binghamiae* powder in distilled water, filtering through a 0.2 μM membrane, and then using the filtrate for experiments.

### 4.4. NOX Activity, iNOS-Derived NO Production, and XO Activity Assays in LPS-Stimulated RAW 264.7 Macrophages

RAW 264.7 murine macrophages were seeded at 1 × 10^5^ cells/well and primed with LPS (0.5 µg/mL, 12 h). Cells were pretreated for 4 h prior to LPS stimulation with EB-WE (100 or 200 µg/mL), pyropheophorbide A (10 µM; sc-264178A, CAS 24533-72-0, Santa Cruz Biotechnology, Dallas, TX, USA), or loliolide (10 µM; SMB01001, CAS 5989-02-6, Sigma-Aldrich, St. Louis, MO, USA). Pathway-selective inhibitors were included as positive controls: APX-115 (10 µM, NOX inhibitor), 1400 W (100 µM, iNOS inhibitor), and allopurinol (100 µM, XO inhibitor). NOX activity was measured from cell lysates using a superoxide-based colorimetric assay kit (E-BC-K815-M, Elabscience, Houston, TX, USA). iNOS activity was assessed indirectly by quantifying nitric oxide (NO) accumulation in culture supernatants using the Griess reagent method. XO activity was determined using an inhibitor screening kit (E-BC-D019, Elabscience, Houston, TX, USAE); The percentage inhibition values provided by the assay were converted to relative activity values using the formula: relative activity (%) = 100 − % inhibition, where lower inhibition corresponds to higher residual XO activity. This conversion was applied to enable direct comparison across treatment groups. All assays were performed in triplicate (*n* = 3), and the results are expressed as mean ± SEM.

### 4.5. ELISA Analysis

Culture supernatants from BMDMs were collected to quantify IL-1β and IL-18 levels using ELISA. After centrifugation to remove cellular debris, cytokine concentrations were measured using Mouse IL-1β/IL-18 DuoSet ELISA kits (R&D Systems, Minneapolis, MN, USA) following the manufacturer’s protocol. Absorbance was read at 450 nm with a microplate reader, and concentrations were calculated based on standard curves.

For intracellular redox cofactor analysis, NAD+/NADH and NADP+/NADPH levels were quantified using WST-8-based colorimetric assay kits (NAD+/NADH: Elabscience, Cat# E-BC-K804-M; NADP+/NADPH: Elabscience, Cat# E-BC-K803-M). BMDMs were lysed in extraction buffer provided by the manufacturer, and supernatants were used for enzymatic cycling reactions followed by WST-8 colorimetric detection. Absorbance was measured at 450 nm using a microplate reader. The resulting values were normalized to the mean ratio of the Normal group (set as 1.0) to allow comparison across groups.

### 4.6. Immunofluorescence (Confocal Microscopy)

For visualization of NLRP3 inflammasome complex formation, BMDMs were seeded at 2 × 10^5^ cells/well in 8-well chamber slides and subjected to the same treatments (LPS + ATP ± EB-WE pretreatment). After stimulation, cells were fixed with 4% paraformaldehyde for 15 min, washed with PBS, and permeabilized with 0.1% Triton X-100. Non-specific binding was blocked with 1% BSA for 30 min. Cells were incubated overnight at 4 °C with primary antibodies against NLRP3 (Abcam, Cambridge, UK) and either ASC or caspase-1 p20 (Cell Signaling Technology, Danvers, MA, USA). The next day, Alexa Fluor-conjugated secondary antibodies were applied for 1 h at room temperature. Nuclei were counterstained with DAPI. Slides were mounted and examined by confocal laser scanning microscopy. Inflammasome speck formation (co-localization of NLRP3 with ASC or caspase-1) was assessed in at least five random fields per group.

In parallel, a separate set of BMDMs was used to visualize the spatial overlap between ROS and NLRP3. After treatment, cells were incubated with 10 µM DCF-DA in serum-free medium for 30 min at 37 °C to detect intracellular ROS. Cells were then washed, fixed with 4% paraformaldehyde, permeabilized, and subjected to immunostaining for NLRP3 (Abcam) overnight at 4 °C. Alexa Fluor 594-conjugated secondary antibodies were applied for 1 h at room temperature, and nuclei were counterstained with DAPI. Confocal images were acquired using a Zeiss LSM confocal microscope (Carl Zeiss, Oberkochen, Germany), and the co-localization of green (ROS) and red (NLRP3) signals was analyzed in five random fields per group.

### 4.7. Western Blot Analysis

To analyze the expression of inflammatory, redox, and barrier-related proteins in BMDMs and colon tissue, Western blotting was performed. For in vitro analysis, BMDMs were stimulated with LPS (1 µg/mL, 5 h) and ATP (5 mM, 30 min), with or without EB-WE pretreatment (100 or 200 µg/mL). After treatment, whole-cell lysates were prepared using RIPA buffer containing protease and phosphatase inhibitors, and culture supernatants were concentrated using TCA precipitation. For in vivo analysis, colon tissue was homogenized in RIPA buffer after DSS treatment and EB-WE administration. Protein concentrations were quantified using a BCA assay, and 20 μg of protein per lane was loaded onto SDS-PAGE gels and transferred to PVDF membranes. Membranes were blocked with 5% skim milk or 5% BSA (for phospho-proteins) for 1 h at room temperature and incubated overnight at 4 °C with the following primary antibodies:

Inflammatory signaling: pro-IL-1β, cleaved IL-1β (p17), pro-caspase-1, cleaved caspase-1 (p10), ASC, iNOS, p-p65 (Ser536), total p65, p-IKBα, IKBα, p-p38 MAPK, total p38 MAPK.

Redox markers: NOX2, NOX4, TXNIP, NQO1, HO-1, NRF2, NADK.

Apoptosis markers: BAX, BCL2, pro-caspase-3, cleaved caspase-3.

Barrier-related proteins: Occludin, ZO-1.

After washing, membranes were incubated with HRP-conjugated secondary antibodies for 1 h at room temperature. Protein bands were detected using enhanced chemiluminescence (ECL) reagents and visualized using a chemiluminescent imaging system. Densitometric analysis was performed using ImageJ software (version 1.54p, National Institutes of Health, Bethesda, MD, USA). Band intensities were normalized to β-actin and expressed as fold change relative to the control group. All Western blot experiments were performed in triplicate (*n* = 3), and results are presented as mean ± standard deviation (SD).

### 4.8. In Vivo Induction of DSS-Induced Colitis and EB-WE Administration

Eight-week-old male C57BL/6 mice (20–22 g) were used in this study. Animals were randomly assigned to four groups (*n* = 10 per group): normal control, DSS control, EB-WE 2 mg group, and EB-WE 5 mg group. To induce colitis, dextran sulfate sodium (DSS; molecular weight, ~36–50 kDa; MP Biomedicals, Irvine, CA, USA) was dissolved in drinking water at a concentration of 2.5% (*w*/*v*) and administered ad libitum for 5 days. The DSS solution was then replaced with regular drinking water for an additional 2-day recovery period, resulting in a total of 7 days of acute colitis induction. In the EB-WE treatment groups, EB-WE was administered by oral gavage once daily, starting one day prior to DSS exposure and continuing throughout the DSS treatment period. EB-WE was suspended in 0.2 mL PBS at doses of 2 mg/day or 5 mg/day. Mice in the normal control and DSS control groups received the same volume of PBS only.

### 4.9. Clinical Monitoring and Disease Activity Index (DAI)

Body weight was measured daily, and stool consistency as well as the presence of fecal blood were monitored throughout the experimental period to assess colitis progression. The disease activity index (DAI) was calculated by summing scores for weight loss, stool consistency, and bleeding. Weight loss was scored as follows: 1–5% loss = 1 point; 5–10% = 2 points; 10–20% = 3 points; >20% = 4 points. Stool consistency was scored as follows: normal firm stool = 0; slightly soft = 1; semi-formed (incipient diarrhea) = 2; complete diarrhea = 3. Bleeding was scored as: no visible blood = 0; mild blood = 1; overt blood = 2; rectal bleeding = 3. The DAI for each mouse was calculated daily, and group means were determined.

### 4.10. Sample Collection and Analysis

On day 7 of DSS administration, all mice were euthanized, and blood and colon tissues were collected. Whole blood was centrifuged at 3000 rpm for 15 min at 4 °C to obtain serum. Serum cytokine concentrations were measured using commercial ELISA kits (R&D Systems) for TNF-α, IFN-γ, IL-2, and IL-4, and all samples were analyzed in duplicate to determine mean values. Colon segments (~1 cm in length) were fixed in 10% neutral-buffered formalin for histological analysis, while the remaining colon tissue was homogenized and processed for Western blot as described in Section 4.5. Fixed colon tissues were embedded in paraffin, sectioned at 7 μM thickness, and stained with hematoxylin and eosin (H&E). Stained slides were examined under a microscope to assess the degree of mucosal injury and inflammatory cell infiltration, and representative pathological findings were documented by photomicrography.

### 4.11. Ethical Approval

All animal experiments were approved by the Institutional Animal Care and Use Committee (IACUC) of Konyang University (Approval No. 24-19-A-08). All procedures were conducted in accordance with the guidelines of the National Institutes of Health Guide for the Care and Use of Laboratory Animals (8th edition), and efforts were made to minimize animal suffering throughout the study.

### 4.12. Statistical Analysis

All data are expressed as mean ± standard deviation (SD). Statistical analyses were performed using GraphPad Prism 9 (GraphPad Software, Boston, MA, USA) and SAS software version 9.4 (SAS Institute Inc., Cary, NC, USA). Student’s *t*-test was applied for comparisons between two groups, and one-way analysis of variance (ANOVA) followed by Tukey’s post hoc test was used for multiple group comparisons. For time-dependent variables such as body weight and disease activity index (DAI), repeated-measures two-way ANOVA was performed to evaluate treatment effects. A *p*-value < 0.05 was considered statistically significant. In all figures and tables, statistical significance is indicated using the following symbols: Asterisks (*, **, ***) indicate comparisons versus the LPS/ATP-treated group (for in vitro data) or the DSS-treated group (for in vivo data). The levels of significance are denoted as follows: * *p* < 0.05; ** *p* < 0.01; *** *p* < 0.001. Daggers (†, ††, †††) indicate comparisons versus the normal untreated control group.

The levels of significance are denoted as follows: † *p* < 0.05; †† *p* < 0.01; ††† *p* < 0.001.

## Figures and Tables

**Table 1:** Putative identification of compounds from EB-WE by UHPLC-QTOF-MS/MS coupled with DB and molecular networking analysis.

Identified Compounds	Monoisotopic Mass (Da)	Formula	Observed Mass (Da)	Observed *m*/*z*	Mass Error (mDa)	Mass Error (ppm)	RT (min)	Detector Counts	Adducts	Score	MS Fragments
ES+ (Positive Ionization Mode)											
Pyropheophorbide A	534.2631	C_33_H_34_N_4_O_3_	534.2627	535.2699	−0.4	−0.8	22.44	556,053	+H, +Na	0.875	223.12, 355.19, 435.25, 447.22
Digiprolactone (Loliolide)	196.1099	C_11_H_16_O_3_	196.11	197.1173	0.1	0.5	6.43	102,239	+H, +Na	0.906	105.12, 133.1, 179.11
Adenosine	267.0968	C_10_H_13_N_5_O_4_	267.0968	268.1041	0.1	0.2	1.36	86,691	+H, +Na	0.98	136.06
Allitol	182.079	C_6_H_14_O_6_	182.0789	205.0681	−0.1	−0.6	0.57	35,257	+Na	0.736	111.05, 182.09
6α-Acetoxy-5-epilimonin	542.2516	C_30_H_38_O_9_	542.2542	565.2434	2.6	4.6	22.8	26,387	+Na	0.85	256.2, 268.2, 503.2
3-Tert-butyl-4-methoxyphenol	180.115	C_11_H_16_O_2_	180.1151	181.1224	0.1	0.3	10.43	25,850	+H, +Na	0.78	108, 137.2, 166.8
ES- (Negative Ionization Mode)											
Monogalactosyldiacylglycerol	766.4867	C_42_H_70_O_12_	766.4898	765.4825	3.1	4	27.89	20,566,572	−H	0.89	225, 317, 537.3
Putative galactolipid (MGDG/DGDG-related)	764.4711	C_42_H_68_O_12_	764.4747	763.4675	3.7	4.8	25.55	1,220,709	−H	0.86	125, 303.2, 561.2
Vachanic acid methyl ester	266.1882	C_16_H_26_O_3_	266.1883	311.1865	0.1	0.3	12.2	161,942	+HCOO	0.82	155.1, 247.2, 267.2
18:3 Lyso-MGDG	598.3142	C_34_H_46_O_9_	598.3114	597.3041	−2.8	−4.7	17.44	163,431	−H	0.86	153, 281.2, 481.2
2-Pentadecanone	226.2297	C_15_H_30_O	226.2295	271.2277	−0.2	−0.7	19.23	103,008	+HCOO, −H	0.65	221.2, 225.2
Helveticoside	534.6527	C_29_H_42_O_9_	579.2838	579.2838	2.7	4.7	16.75	88,100	+HCOO	0.77	225, 279.23, 319.23

Abbreviations: RT, retention time; Da, Dalton; ppm, parts per million.

## Data Availability

The original contributions presented in this study are included in the article/Supplementary Material. Further inquiries can be directed to the corresponding authors.

## Supplementary Materials

The following supporting information can be downloaded at https://www.mdpi.com/article/10.3390/ijms27062674/s1.

## Abbreviations

The following abbreviations are used in this manuscript:

AP1	Activator Protein 1
ASC	Apoptosis-associated speck-like protein containing a CARD
ATP	Adenosine Triphosphate
BAX	Bcl-2-associated X protein
BCL2	B-cell lymphoma 2
BMDM	Bone Marrow-Derived Macrophage
CAT	Catalase
COX2	Cyclooxygenase 2
CaMKII	Ca^2+^/Calmodulin-dependent Protein Kinase II
Caspase-1	Cysteine-aspartic protease 1
DAI	Disease Activity Index
DAMPs	Damage-Associated Molecular Patterns
DCFDA	2′,7′-Dichlorodihydrofluorescein diacetate
DSS	Dextran Sulfate Sodium
EB-WE	*Endarachne binghamiae* Water Extract
ERK	Extracellular signal-Regulated Kinase
GRX	Glutaredoxin
GSH	Glutathione
IBD	Inflammatory Bowel Disease
IFN-γ	Interferon gamma
IL-18	Interleukin-18
IL-1β	Interleukin-1 beta
IκBα	Inhibitor of kappa B alpha
JNK	c-Jun N-terminal Kinase
LPS	Lipopolysaccharide
MAPK	Mitogen-Activated Protein Kinase
MMP9	Matrix Metalloproteinase 9
NADK	NAD + kinase
NADPH	Nicotinamide Adenine Dinucleotide Phosphate (reduced form)
NF-κB	Nuclear Factor kappa-light-chain-enhancer of activated B cells
NLRP3	NACHT, LRR and PYD domains-containing protein 3
NOX	NADPH Oxidase
NQO	NAD(P)H Quinone Dehydrogenase
ONOO^−^	Peroxynitrite
PAMPs	Pathogen-Associated Molecular Patterns
PRDX	Peroxiredoxin
ROS	Reactive Oxygen Species
RelA	v-rel avian reticuloendotheliosis viral oncogene homolog A
SOD	Superoxide Dismutase
SREBP2	Sterol Regulatory Element-Binding Protein 2
TLR4	Toll-like Receptor 4
TNF-α	Tumor Necrosis Factor alpha
TRX	Thioredoxin
TUNEL	Terminal deoxynucleotidyl transferase dUTP nick end labeling
TXNIP	Thioredoxin-interacting protein
UHPLC-QTOF-LC-MS/MS	Ultra-High Performance Liquid Chromatography–Quadrupole Time-of-Flight Liquid Chromatography–Mass Spectrometry/Mass Spectrometry
iNOS	inducible Nitric Oxide Synthase
p38	p38 Mitogen-Activated Protein Kinase
